# Host–Parasite interactions in *Entamoeba histolytica* and *Entamoeba dispar*: what have we learned from their genomes?

**DOI:** 10.1111/j.1365-3024.2011.01325.x

**Published:** 2012-02

**Authors:** I W Wilson, G D Weedall, N Hall

**Affiliations:** Institute of Integrative Biology, University of LiverpoolCrown Street, Liverpool, UK

**Keywords:** differential gene expression, *Entamoeba*, genome, virulence

## Abstract

Invasive amoebiasis caused by *Entamoeba histolytica* is a major global health problem. Virulence is a rare outcome of infection, occurring in fewer than 1 in 10 infections. Not all strains of the parasite are equally virulent, and understanding the mechanisms and causes of virulence is an important goal of *Entamoeba* research. The sequencing of the genome of *E. histolytica* and the related avirulent species *Entamoeba dispar* has allowed whole-genome-scale analyses of genetic divergence and differential gene expression to be undertaken. These studies have helped elucidate mechanisms of virulence and identified genes differentially expressed in virulent and avirulent parasites. Here, we review the current status of the *E. histolytica* and *E. dispar* genomes and the findings of a number of genome-scale studies comparing parasites of different virulence.

## Introduction

Amoebiasis is a disease of global importance, caused by the eukaryotic parasite *Entamoeba histolytica*. It is the most common worldwide cause of mortality from a protozoon after malaria, killing an estimated 40 000–110 000 people annually, and causing 34–50 million cases of severe disease. However, fewer than 10% of those infected develop invasive amoebiasis (1). Those most at risk are people living in areas of poor sanitation, as the parasite is transmitted via a faecal–oral route. In such environments, exposure may be very high. For example, acquisition of *Entamoeba*-specific antibodies indicated an annual incidence of infection of 40% in children living in a slum in Bangladesh (2). In Hué, Vietnam prevalence of Amoebic Liver Abscesses (ALA) was higher in a more densely populated area than in the city as a whole (3,4). In more affluent countries, where poor living conditions are less common, amoebiasis tends to be seen in certain groups, such as travellers returning from endemic areas (5), men who have sex with men and institutionalised individuals (6–9). Heterosexual and female homosexual activity can also transmit amoebiasis (10). Overall, men are more susceptible to invasive amoebiasis than women, despite similar infection rates (11). It is hypothesised that, in pathogenic *E. histolytica* infections, resistance to invasion is determined by a relatively small number of host genes (12).

The molecular biology of *Entamoeba* is complex, and much remains unknown, including chromosome number, ploidy and whether they undergo sexual reproduction. In an effort to better understand the biology of *E. histolytica*, its genome was sequenced along with that of the related species *Entamoeba dispar*. Since the first assembly and annotation of the *E. histolytica* genome in 2005 (13,14), significant advances have been made in understanding host–parasite interactions and virulence in *Entamoeba*. In this review, we describe the current status of genome annotation in virulent and nonvirulent *Entamoeba* species and review some of the important genes identified by genomic, proteomic and transcriptomic studies in the context of the pathogenic *E. histolytica* life cycle.

### *Entamoeba histolytica*'s pathogenic life cycle

**Figure 1:**
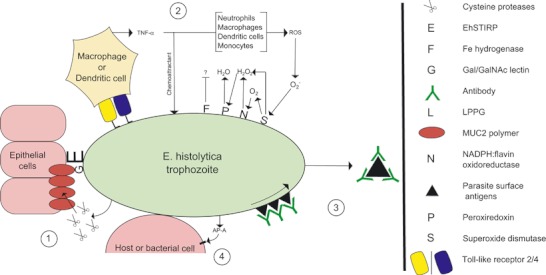
Key virulence factors of *Entamoeba histolytica* involved in pathogenic infections that have been identified by genome-scale investigations. 1 = Binding to epithelial extracellular matrix via Gal/GalNac lectin and EhSTIRP; and degradation of MUC2 polymers via secreted cysteine proteases. 2 = Subversion of host immune response, following binding of LPPG to host Toll-Like receptors 2 and 4, via degradation of reactive oxygen species by superoxide dismutase, NADPH:flavin oxidoreductase and peroxiredoxin. Fe-hydrogenase inhibits immune response by unknown mechanism. 3 = ‘Capping and Shedding’ of trophozoite surface antigens by host antibodies and lectins, involving cytoskeletal rearrangement to translocate antigen–antibody complexes to ‘uroid’ of cell for shedding. Putative function for EhROM1 in translocation. 4 = Direct contact between trophozoite and host or bacterial cell, leading to secretion of amoebapore-A, which forms pores in target cell membrane without need for receptor.

*Entamoeba histolytica* has a two-stage life cycle, existing as resistant infective cysts in the environment and potentially pathogenic trophozoites in the human colon. Upon excystation, trophozoites follow one of two paths. The more common path is commensal colonisation, where trophozoites inhabit the gut lumen and feed on enteric bacteria by phagocytosis, a process involving rearrangement of the amoebic cytoskeleton to internalise bacteria in lytic phagosomes (15). The less common path leads to invasive amoebiasis. Virulence factors allow the parasite to cause pathogenic amoebiasis via a variety of mechanisms, crucially including those that allow it to resist and subvert the host's innate and adaptive immune responses (Figure 1). Upon activation, previously commensal trophozoites degrade the colonic mucosal layer then bind to host epithelial cells (16,17). As reviewed by Lejeune *et al.* (18), the bound trophozoites trigger pathology in the host tissues, promoting penetration and infection. Apoptosis is induced in the trophozoite-bound epithelial cells as a result of cascading secretory proinflammatory cytokines. This cellular damage and the subsequent lateral invasion through the submucosa result in tissue inflammation and characteristic flask-shaped ulcers (19). The importance of apoptosis in amoebic virulence (20) is highlighted by studies on the leptin signalling pathway. Leptin signalling has multiple roles in the human body including regulation of the immune response to infection (towards a Th1 inflammatory response) and preventing apoptosis; however, experiments in mice show that it is leptin's anti-apoptotic role in gut epithelia, rather than its role in immune effector cells, which mediates susceptibility (21). An amino acid substitution (glutamine to arginine) in the leptin receptor is associated with increased susceptibility to, and severity of, infection in both mice and humans (22).

In many respects, the immune response to *E. histolytica* infection resembles that raised against the intestinal parasites *Cryptosporidium* and *Giardia* (23,24), with important roles for reactive oxygen species (ROS), nitric oxide (NO) and secreted IgA (25,26). Host immunity and pathology are closely linked. Human immune cells are recruited to the site of trophozoite invasion and, whilst attacking trophozoites, enhance the pathology caused by the invasion. NO and ROS released by immune effector cells damage *E. histolytica* trophozoites; however, the parasites have evolved means to minimise damage caused by these oxygen species, including the expression of various surface molecules (27–31) and internalisation and destruction of host immune cells (as well as other host cells) by phagocytosis (15).

*Entamoeba histolytica* also faces challenges from adaptive immunity. Adaptive immunity appears to protect against symptomatic disease, although not reinfection (32,33). The occurrence of subsequent infections indicates that immunity is either incomplete, ineffective against heterologous parasite strains or that the parasite utilises effective immune evasion strategies. For example, immunoglobulins binding to surface proteins may block adhesion and activate the complement pathway. Trophozoites appear to be able to evade this arm of immunity by a process of ‘capping and shedding’ where bound antibodies are moved to the rear of the trophozoite, forming an ‘uroid’, and are shed. The host immune system is temporarily ‘blind’ to the parasite until different surface receptors are bound, at which point the process begins again (34,35).

Trophozoites that penetrate and cross the intestinal epithelium can be disseminated to other organs, most commonly the liver, where they form abscesses. Entering the relatively oxygen-rich environment of the bloodstream exposes the trophozoites to greater oxidative stress. In addition, greater exposure to humoral immunity and the complement system places the trophozoites at greater risk of inhibition and degradation. Consequently, it is likely that trophozoites require different molecular pathways to cause ALA, rather than remain as intestinal infections (36,37).

In support of this theory, virulent *E. histolytica* trophozoites exposed to conditions inducing heat shock demonstrate differential gene expression. According to a microarray analysis of 1131 transcripts, 471 genes were downregulated and 40 upregulated when cells grown at 37°C were incubated at 42°C for 4 h. It has been hypothesised that the large number of downregulated genes is indicative of a general molecular reaction to a heat shock-induced homeostatic imbalance (38).

After entering the hepatic sinusoids, pathogenic trophozoites invade the parenchyma. The hepatocytes and trophozoites are physically separated by a barrier of polymorphonuclear leukocyte (PMNs) and mononuclear host cells. The trophozoites make direct contact with the PMNs, resulting in lysis of the immune cells and the release of their own lytic enzymes, which damage surrounding hepatocytes. As surviving trophozoites reproduce and spread, the necrotic regions coalesce into abscesses. Immune epithelioid cells segregate these regions from healthy tissue, forming granulomas, in which the trophozoites are trapped with the expanding necrotic zones (37,39).

### Differential virulence between *Entamoeba* species and strains

**Figure 2:**
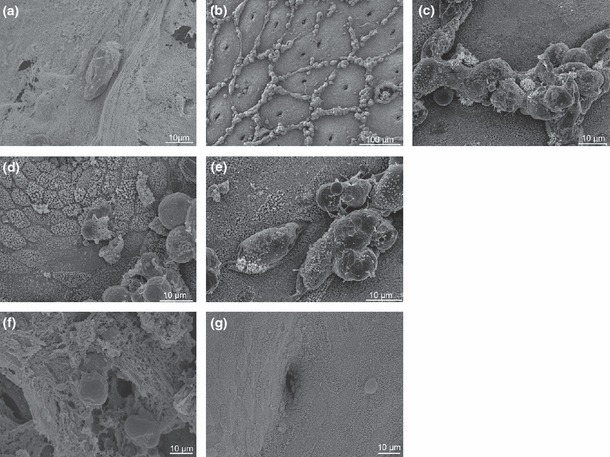
Comparison of colonisation of the colonic surface by *Entamoeba histolytica* and *Entamoeba dispar*. Panels show breakdown of mucus by *E. histolytica* after 0 h (a) and 2 h (b). Enlargement of region shows aggregates of trophozoites and recruited human cells (c). After 4 h, trophozoites begin to damage (d) and to penetrate epithelia (e). Conversely, after 4 h, *E. dispar* binds to, but does not degrade, the mucus barrier (f) and, as shown by manually removing the mucus layer, does not recruit immune cells to the epithelial surface (g). [Reprinted, with permission, from (44)].

Evidently, not all *E. histolytica* strains are equally virulent. The genomic reference strain, *E. histolytica* HM-1:IMSS, is the best-studied virulent strain of *E. histolytica*, derived from a colonic biopsy taken from a man with dysentery in Mexico in 1967 (13,40). The *E. histolytica* Rahman strain was isolated from the stool of an Indian sailor in the UK in 1964 (41) and is considered to be avirulent. It has reduced ability to phagocytose erythrocytes, damage colonic epithelia and cause ALA, relative to HM-1:IMSS (29,42). A close human-infective relative of *E. histolytica* is *E. dispar*, which is morphologically indistinguishable from *E. histolytica* by microscopic analysis. Only in 1993 was it described as a distinct species, under the name ‘*dispar’* originally used by Brumpt in 1925 (43). *E. dispar* is avirulent. Tracking *E. dispar* (strain SAW1734) cells on human colonic explants shows that they do not break down the mucus barrier or cause epithelial cell damage, unlike *E. histolytica* HM-1:IMSS (Figure 2) (44). Recently, however, *E. dispar* has been associated both with cases of amoebic colitis and ALA, and its avirulence status has been questioned (45).

### The genomes of *Entamoeba histolytica* and *Entamoeba dispar*

The draft assembly and annotation of the *E. histolytica* HM-1:IMSS genome was published in 2005 (13,14). A reassembly of the genome, including more sequence data and new annotation, was published in 2010 (46). The genome assembly and annotation was held on the Pathema website (http://pathema.jcvi.org/Pathema/) (47). More recently, the data have been made available on AmoebaDB (http://www.amoebadb.org), part of the EuPathDB web resource (48–50), along with the as-yet unpublished genome sequence of *E. dispar*. The *E. histolytica* genome assembly represents approximately 20 Mb of sequence, covered to >12.5 × depth (13,46). It remains fragmentary, comprising 1496 scaffolds, most likely due to the high number of repetitive elements in the genome (51). The *E. dispar* assembly is slightly larger than that of *E. histolytica* (∼22 Mb), but is sequenced to lower coverage depth (∼4.5×) and is more fragmentary (3312 scaffolds). A total of 8745 genes are predicted, slightly more than the ∼8300 for *E. histolytica*. Average divergence between orthologous genes of the two species is approximately 38% at synonymous sites (Weedall G., unpublished observations).

**Figure 3:**
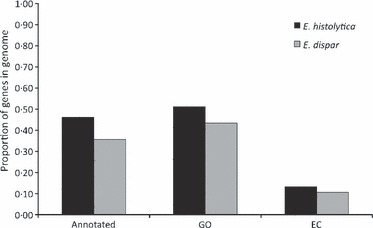
Comparison of the current status of the *Entamoeba histolytica* and *Entamoeba dispar* genome annotations, indicating the relative proportions of genes with putative functions. ‘Annotated’ = Percentage of non-hypothetical genes in the annotation; ‘GO’ = Percentage of genes associated with a ‘Gene Ontology’ term, i.e. those with either a cellular component, molecular function or biological process; ‘EC’ = Percentage of genes with ‘Enzyme Commission’ numbers, i.e. enzymes identified as being involved in known chemical reactions. Based upon figures from AmoebaDB (48–50). Based on most recent genome annotation (46).

The reassembly and reannotation of the *E. histolytica* genome reduced the estimated number of genes from ∼10 000 to 8333, largely because of the removal of apparently artefactual paralogues, very short gene models and truncated genes (46). The majority of genes (∼55%) encode unknown proteins (Figure 3). This can be compared to other gut parasites, the apicomplexan *Cryptosporidium parvum* (40% of 4367 genes are annotated as hypothetical) and the diplomonad *Giardia lamblia* (75% of 9747 genes are annotated as hypothetical in isolate WB from assemblage A) (data from EuPathDB). The predominance of uncharacterised genes presents a problem for genome-wide analyses because the majority of genes of interest are often of unknown function. The facility to upload corrected annotations to the genome is available (47,49), and such ‘community annotation’ has been encouraged (52). Researchers can post corrections to gene models, links to validating data and functional annotations that can be incorporated into future annotations. Annotation of hypothetical proteins in other species, such as *Plasmodium falciparum*, has been improved by using annotated genes with similar transcriptional profiles, annotated orthologues and automated literature mining (53). Similar methods may aid the annotation of *E. histolytica*.

### Genome-scale analyses of *Entamoeba* virulence

*Entamoeba* genome sequences are used either as a means to identify sequences generated by processes such as mass spectrometry of peptides (29,54,55) or sequencing of cDNA from differential display PCR (36,56), or to design microarrays for hybridisation-based analyses (57–61). Many genes involved in amoebic pathogenesis have been identified by genome-wide analyses. Investigations have compared gene expression in the same strain in different environments, identifying genes that may be important for survival in these environments (36,59), and compared cell lines that show different virulence characteristics (29,57,58,60,62).

A DNA microarray created from a clone library representing 2110 unique genes has been used to compare diversity of genomic DNA among *E. histolytica* and *E. dispar* strains (63) and transcriptional differences between *E. histolytica* HM-1:IMSS, *E. histolytica* Rahman and *E. dispar* SAW760 (60). A 70-bp oligonucleotide DNA microarray representing 6242 unique *E. histolytica* HM-1:IMSS genes has been used to compare transcriptional differences between HM-1:IMSS and Rahman and to compare syngenic cell lines of differential virulence derived from HM-1:IMSS (57,58). A different microarray using 25-bp oligonucleotide probes representing 9435 *E. histolytica* HM-1:IMSS open reading frames has been used to compare *E. histolytica* trophozoites from murine intestinal infections and from *in vitro* culture (59) and to compare the transcriptional responses of HM-1:IMSS and Rahman to oxidative and nitrosative stress (61).

The numbers of putative differentially expressed genes among strains vary with the different methods and criteria used to define differential expression. However, broad trends are apparent. A greater proportion of *E. dispar* genes than Rahman genes appear to be downregulated relative to HM-1:IMSS (58,60). A number of genes are downregulated in both avirulent cell lines. For instance, of 32 genes with lower mRNA expression in Rahman, 29 also showed lower expression in *E. dispar* (60). The following sections describe some of the genes identified by these studies as potentially important virulence factors.

#### Genes involved in survival and virulence in the intestine

Experimental infections of mouse intestines induced differential expression (twofold or greater) of 523 genes: 326 on day 1 post-infection, 109 on day 29 post-infection and 88 at both time points (59). The authors speculated that an initial stress response associated with adaptation to the new environment might partly explain the large number of genes differentially regulated early in infection. Among putative virulence factors showing differential expression were cysteine proteases and members of the galactose- and *N*-acetyl-_D_-galactosamine-binding lectin (Gal/GalNAc–lectin) complex on the parasite surface (16).

An important process in amoebic virulence is the degradation of the mucus layer, which enables the trophozoites to reach the gut epithelial layer (Figure 2). Trophozoites release cysteine proteases to degrade the main component of the mucus barrier, MUC2. Different members of the cysteine protease gene family are expressed in culture and in mouse intestine, suggesting that different gene family members may play unique roles important in different environments (59). Cysteine protease expression is lower overall in *E. dispar* than in *E. histolytica* (64), indicating their role in virulence, and CP-A5 (gene ID, EHI_168240), a key protease for the degradation of the MUC2 polymer (17,65,66), is a pseudogene in *E. dispar* (67). However, CP-A5 showed no statistically significant differential expression between *E. histolytica* HM-1:IMSS and Rahman. CP-A4 (EHI_050570), CP-A6 (EHI_151440) and CP-B1 (EHI_117650) were expressed to a greater degree in HM-1:IMSS, whereas CP-A3 (EHI_159610), CP-A7 (EHI_039610) and CP-B9 (EHI_181230) were greater in Rahman (58). Numerous cysteine protease genes (e.g. EHI_127470, EHI_019390, EHI_144040 and EHI_132640) are pseudogenes in the *E. histolytica* genome, and this, along with their divergence from *E. dispar* orthologues (64), suggests a degree of evolutionary plasticity in this gene family.

Trophozoites bind to the mucus layer and to epithelial cells via the Gal/GalNAc–lectin complex (16). Two genes encoding light subunits in the Gal/GalNAc lectin – *lgl*2 (EHI_049690) and *lgl3* (EHI_027800) – were downregulated to different degrees during the course of intestinal infection (59). The importance of the downregulation of *lgl3* in invasive infection was supported by transcriptional analysis showing 22-fold higher expression in the nonvirulent Rahman strain, compared with HM-1:IMSS (58). Other molecules involved in binding to host cells include the *E. histolytica* serine-, threonine- and isoleucine-rich proteins (EhSTIRP) (68). These proteins are encoded by a small gene family in *E. histolytica* (EHI_012330, EHI_004340 and EHI_025700).

#### Surviving host responses to invasive amoebiasis

Nitric oxide and ROS released by neutrophils, macrophages, monocytes and dendritic cells constitute a major threat to the trophozoites, which they can counteract by the actions of a number of molecules expressed on their surfaces: peroxiredoxin, superoxide dismutase (SOD) and NADPH:flavin oxidoreductase (27–29,31). SOD generates H_2_O_2_ in the presence of O_2_^−^, NADPH:flavin oxidoreductase catalyses the reduction of O_2_ to H_2_O_2_, and peroxiredoxin reduces the H_2_O_2_ from both pathways to H_2_O (69). Fe-hydrogenase, which, in bacteria, is involved in survival of oxidative stress (30), is also expressed by the trophozoites (58). Large numbers of genes show differential regulation in response to oxidative and nitrosative stress, and there is a substantial overlap in the genes involved in these responses. HM-1:IMSS shows a more robust response to stress than Rahman, with more genes differentially regulated overall and to a greater degree (61).

Peroxiredoxin is more highly expressed in *E. histolytica* HM-1:IMSS than in Rahman according to analyses of protein (29) and mRNA (60) expression. Furthermore, it is downregulated in *E. dispar* relative to *E. histolytica* HM-1:IMSS (28,60). In *E. histolytica* HM-1:IMSS, peroxiredoxin is expressed on the surface where it is co-localised with the Gal/GalNAc lectin, possibly to degrade ROS released from bound immune cells (28). In contrast, expression in *E. dispar* is restricted to the cytoplasm, suggesting an inability of *E. dispar* to survive the oxidative burst that would be inflicted upon it following host invasion. *E. histolytica* peroxiredoxin sequences are highly divergent from their *E. dispar* orthologues (63). The current *E. histolytica* genome annotation contains a number of putative peroxiredoxin genes (EHI_001420, EHI_061980, EHI_114010, EHI_122310, EHI_123390, EHI_201250, EHI_145840, EHI_018740, EHI_183180 and EHI_084260) and pseudogenes (EHI_121620, EHI_139570 and EHI_172720). Whether all of these genes are real, functional and expressed remains to be determined. If so, it is possible that gene copy number variations between strains and species of *Entamoeba* affect overall gene expression levels.

Involvement of other putative oxidative stress response genes in virulence is less clear. Fe-hydrogenase (EHI_073390) is more highly expressed in *E. histolytica* HM-1:IMSS than in *E. dispar*, suggesting a role in virulence, yet within *E. histolytica*, it is more highly expressed in Rahman than HM-1:IMSS (58,60). SOD (EHI_159160) is also more highly expressed in *E. histolytica* Rahman than in HM-1:IMSS (29). SOD does appear to play a role in oxidative stress resistance: increased expression of SOD and peroxiredoxin is associated with metronidazole resistance, implying an involvement in detoxification of nitrogen-based free radicals generated by metronidazole activation (70,71).

Immunoglobulins binding to amoebic surface proteins can disrupt trophozoite cell functions, block adhesion to host receptors and activate the complement pathway. The parasite can avoid these outcomes by cysteine protease-mediated clipping of bound antibodies and complement (72,73) and by shedding the bound antibodies from its surface. Binding of host antibodies to amoebic surface antigens induces actin- and myosin-mediated redistribution to a membranous posterior appendage of the cell, the ‘uroid’, where this ‘cap’ is shed mostly as membrane-bound vesicles (34,35,74). A rhomboid protease involved in shedding surface proteins, EhROM1 (EHI_197460), was identified by searching the *E. histolytica* genome sequence for motifs conserved across known rhomboid proteases (75). EhROM1 specifically cleaves the heavy chain subunit of the Gal/GalNAc lectin and localises to the uroid (75). However, EhROM1 knock-down mutants showed no significant change in cap formation or complement resistance, but did show reduced ability to adhere to host cells and reduced phagocytic ability (76), suggesting a novel role for this protein (75,76). Proteomic analysis of uroid-extruded vesicles identified several surface-linked proteins, in addition to the Gal/GalNAc lectin, that are apparently capped and discarded, implying that they are involved in host–amoeba interactions. These included calreticulin, a multifunctional antigen with a notable involvement in calcium signalling, and the variable surface antigen M17 (77).

A number of proteins with uncertain functions show differential expression between *E. histolytica* HM-1:IMSS and the noninvasive Rahman and *E. dispar* (29,59). Grainin-1 was upregulated in *E. histolytica* Rahman, and grainin-2 was upregulated in both nonvirulent cell lines. The sequences of both grainins contain at least one metal-ligating EH-hand motif, commonly seen in proteins that bind calcium. Both genes are upregulated in culture in response to inducers of programmed cell death (PCD), and a stress-response role diminishing intracellular Ca^2+^ was suggested (78), as was a role in calcium-dependent endocytosis and granular exocytosis, aiding pathology (79). Lower levels of expression of both genes in mouse intestines, and of grainin 1 in ALA samples, were seen relative to *in vitro* cultures, possibly owing to higher stress levels in *in vitro* conditions (36,59). In the current genome assembly, seven putative grainin genes are annotated: grainin 1 (EHI_167300) is relatively divergent from its nearest paralogues (EHI_120360, 71% amino acid identity; EHI_060380, 57% identity); grainin 2 (EHI_167310) has a shorter, near-identical, paralogue (EHI_111720); both are relatively divergent from their nearest paralogues (EHI_164430, EHI_164440, both 56% amino acid identity).

A LIM domain-containing protein (EHI_096420) was more highly expressed in *E. histolytica* HM-1:IMSS than in Rahman or *E. dispar* (29,54). Its function is not known, but it has been shown to localise to the plasma membrane and to bind to the actin cytoskeleton via its LIM domain (80). Alcohol dehydrogenase 3 (ADH3) was more highly expressed in HM-1:IMSS relative to Rahman and *E. dispar* (29,54). ADH3 (EHI_125950 and EHI_198760) is expressed at greater levels on the cell surface of HM-1:IMSS than *E. dispar* and, when overexpressed in HM-1:IMSS and Rahman cells, increased host inflammatory response, although no definite role in virulence was determined (54). ADH2 (EHI_024240, EHI_150490 and EHI_160940) was more highly expressed in HM-1:IMSS than in *E. dispar* (54). ADH2 is associated with the cell membrane and is involved in iron scavenging from the host's transferrin (81).

Trophozoites can phagocytose host epithelial cells, erythrocytes and immune cells. Phagocytosis is modulated by the motor protein myosin IB, which cross-links actin filaments, to restructure the amoebic cytoskeleton as necessary (82). Proteomic analysis, by liquid chromatography and tandem mass spectroscopy (LC-MS/MS), of phagosome proteins allows identification of proteins differentially expressed over time and in different conditions. In wild-type *E. histolytica* HM-1:IMSS and a strain overexpressing myosin IB (MyoIB+), approximately 1000 proteins were identified overall. Of these, about 150 proteins present in the early phagosome were associated with the cytoskeleton (including actin, coactosin and talin), were signalling molecules (including PI3-K and Ras GAP) or were involved in intracellular trafficking (including calreticulin). Of those associated with the cytoskeleton, seven proteins were functionally linked to myosin IB, demonstrable by their expression in detectable levels in MyoIB+ only (83). Also in HM-1:IMSS, of 159 phagosomal proteins detected, 51 were constitutively expressed, whilst the remaining 108 showed differential expression across the monitored 2-h period. Those constitutively expressed included CP-A5, actin and the Gal/GalNAc lectin. The more numerous transient proteins included many Rab GTPases and several of the Rac cytoskeletal proteins, reflecting the necessary fluidity of the cytoskeleton in the phagocytic process. The same study reported inter-strain variation in expressed *E. histolytica* phagosome proteins, suggesting a role in differential virulence (84).

#### Virulence factors involved in amoebic liver abscess

Death from amoebiasis results mainly from the formation of abscesses on the liver after trophozoites escape the gut, so understanding the molecular basis of abscess formation is of considerable interest. Comparisons of the transcriptomes of *E. histolytica* trophozoites axenically cultured *in vitro* with those isolated from liver abscesses using differential display PCR (DD-PCR) identified small numbers of genes differentially expressed between the two (36,56). Among these were genes encoding grainin-1, a flavoprotein, a GTP-binding protein and ribosomal proteins (36,56).

A cell line derived from HM-1:IMSS (‘HM1A’), which has lost the ability to cause ALA, has been compared to virulent HM-1:IMSS (‘HM1B’) at both proteomic and transcriptomic levels (57,62). Eighty-seven genes showed twofold or greater differential (mRNA) expression between HM1A (47 genes upregulated) and HM1B (40 genes upregulated) (57). Thirty-one proteins showed 2.3-fold or greater differential protein expression between HM1A (21 upregulated) and HM1B (10 upregulated) (62). Only two genes, Fe-hydrogenase-2 (EHI_005060) and a C2-domain-containing protein (EHI_069320), were found differentially expressed (upregulated in HM1A) at both the proteomic and the transcriptomic levels. Despite using the same microarray, little overlap was seen in the transcripts downregulated in HM-1:IMSS clone A and in Rahman, relative to HM-1:IMSS clone B (57,58). Only 1 gene was significantly downregulated in both Rahman and avirulent HM-1:IMSS, and of the 152 transcripts upregulated in *E. histolytica* HM-1:IMSS relative to Rahman, only five were also significantly upregulated in the pathogenic HM-1:IMSS clone B relative to clone A. Two of these five genes encoded AIG1-like proteins. AIG1 proteins are small GTPases originally identified in *Arabidopsis thaliana* (85) where they confer resistance to bacterial infections. AIG1-like proteins are encoded by a large gene family in *E. histolytica* (57,59) and may be involved in bacterial interactions. This lack of overlap suggests that the nature of avirulence in Rahman and HM-1:IMSS clone A may be quite different.

Another investigation comparing virulent and avirulent lines derived from the *E. histolytica* HM-1:IMSS strain compared mRNA expression in 1130 genes and showed downregulation (>twofold, *P* < 0.05) of 21 genes and upregulation of 29 genes in the virulent line (86). Among the upregulated genes in the virulent line were the surface antigen ariel-1, which has been shown to be absent from *E. dispar* (87), and several lysine-rich proteins (‘KRiPs’) and lysine- and glutamic acid-rich proteins (‘KERPs’). Gene knock-down of KERP1 using antisense RNA reduced the formation of liver abscesses (86).

None of these studies identified the virulence factor amoebapore-A (AP-A; EHI_159480). The amoebapore's role in pathogenesis has been demonstrated in hamster and severe combined immunodeficient (SCID) mouse livers (88,89). AP-A appeared to be essential for ALA formation in hamsters, but suppression in the mouse model did not completely prevent ALA, suggesting that other processes are important in ALA formation. AP-A is inserted into host plasma membranes, without the need to bind to a host receptor, upon direct contact between a trophozoite and a host cell (90), forming pores and lysing the host cell (91). Amoebapores also have a bacteriolytic function, being able to lyse gram-positive bacteria (88,90).

### Characterising candidate virulence factors

Characterisation of gene function has proven difficult in *Entamoeba* as gene knock-outs have not been achieved. There has, however, been some success with transcriptional gene silencing and, more recently, with RNAi-mediated gene knock-down (92–94). The gene encoding AP-A has been silenced in some, although not all, cell lines, using what was originally designed to be a putative overexpression vector. The mechanism of silencing is not known for certain, although involvement of a short interspersed element (SINE) and of tRNA repeat arrays in the vector have been proposed (95,96). The ‘G3’*E. histolytica* cells this silencing mutation gave rise to are virulence attenuated, being impaired in their ability to digest phagocytosed cells (although not impaired in their ability to phagocytose them in the first instance) and unable to cause ALA (88,97). Cell lines that have been silenced for AP-A expression continued to show AP-A silencing even when selection for the vector was removed, although in other cases, silencing has not been integrated permanently into the cell lineage and future generations have reverted to their wild type (98). Moreover, additional silencing of genes could be achieved in this line using a vector with an additional gene in it. By this method, CP-A5 and *Ehlgl* were silenced (99,100). Gene silencing affected multiple members of the gene families containing the target gene (99), and, interestingly, downregulation of several *lgl* genes led to upregulation of others, a possible compensatory mechanism. Silencing of EhLIM-A – the gene encoding a LIM-like protein – has been achieved in a similar fashion (80). RNAi-mediated gene knock-down has been achieved using different methods of administering the siRNA: bacterial expression of double-stranded RNA followed by either adding the bacteria to *Entamoeba* culture or extracting the dsRNA and soaking *Entamoeba* trophozoites in them (94), or addition of vectors expressing short hairpin RNA (sense and antisense linked by a loop) to the trophozoites (92). Beta-tubulin, KERP1, URE3-BP, IGl and EhC2A have been ‘knocked down’ by these methods. Continued improvement of molecular tools for targeted gene silencing will help to characterise the roles of specific genes and gene families in host–parasite interactions.

### Concluding Remarks

The genome-scale studies made possible by sequencing of the *E. histolytica* genome have greatly improved our knowledge of the pathogenesis of *E. histolytica* and identified many genes that may play important roles in host–parasite interactions. Comparisons of different strains of *E. histolytica* and of the related species *E. dispar* show differences in sequence and in expression that may account for different virulence profiles. In order for further genome-scale studies into genetic and gene expression differences to be successful in the future improved gene annotation is vital. It is hoped that a model of ‘community annotation’ may help rapidly improve and disseminate information characterising *Entamoeba* genes. Much work has yet to be done before we understand the complexities of *Entamoeba* virulence. Continual improvement to the assembly and annotation of *Entamoeba* genomes is central to this effort.
